# Bioorthogonally Cross-Linked Injectable PEG Hydrogel with Robust Hemostatic and Antibacterial Properties

**DOI:** 10.3390/gels12060556

**Published:** 2026-06-20

**Authors:** Jun Zhai, Qiwen Huang, Lei Ni, Chenming Li, Li Hao, Jian Chen, Cheng Chi, Risheng Li, Yong-Miao Shen, Ronggui Lu, Zhijun Zhang

**Affiliations:** 1 Department of Chemistry, School of Chemistry and Chemical Engineering, Zhejiang Sci-Tech University, Hangzhou 310018, China; zhaijun0307@163.com (J.Z.);; 2 Shengzhou Innovation Research Institute of Zhejiang Sci-Tech University, Shengzhou 312400, China; 3 Department of Ultrasound in Medicine, The Fourth Affiliated Hospital of School of Medicine, International School of Medicine, International Institutes of Medicine, Zhejiang University, N1 Shangcheng Avenue, Yiwu 322000, China; 4 Zhejiang Dongbang Pharmaceutical Co., Ltd., Linhai 317022, China

**Keywords:** injectable hydrogel, bioorthogonal chemistry, hemostasis, antibacterial activity, thrombin, PEG, click chemistry

## Abstract

The rapid hemostasis of deep and irregular wounds is of great clinical significance. In this study, an injectable hemostatic hydrogel based on bioorthogonal conjugation was developed. This gel uses thrombin (TMB) as the hemostatic active substance and 4ARM-PEG-N_3_ as the crosslinking agent, which undergo orthogonal conjugation via the classic azide–alkyne click reaction to form an injectable hydrogel (TMB-PEG). The resulting hydrogel exhibited a transparent, injectable gel state. TEM images revealed that the hydrogel comprised sheet-like structures and interwoven fibers with a diameter of approximately 100 nanometers. In a puncture bleeding wound model, hemostasis with the TMB-PEG hydrogel required only 25 s, with a blood loss of 1.9 ± 1.3 mg, both approximately one-sixth of that of the control group. Moreover, the hemostatic performance of the TMB-PEG hydrogel was far superior to that of three other commonly used hemostatic materials. Furthermore, cephalosporin antibiotics were conjugated to the hemostatic gel via orthogonal reactions, endowing it with significant broad-spectrum antibacterial activity, achieving over 99% antibacterial efficacy against both Gram-negative and Gram-positive bacteria.

## 1. Introduction

Uncontrolled hemorrhage remains the leading cause of preventable death in trauma [1,2,3], particularly in scenarios involving deep, irregular, or non-compressible wounds, such as those encountered in traffic accidents, combat injuries, and major surgery [4]. Visceral organs, like the liver and spleen, present an immense hemostatic challenge owing to their complex vasculature and fragile parenchyma [5]. Conventional approaches—electrocautery [6], compression [7], suturing [8], and tourniquets [9]—are often inadequate for massive bleeding from visceral ruptures or penetrating torso trauma and may even exacerbate coagulopathy or induce ischemic injury [10,11,12]. Current hemostatic materials based on the coagulation cascade frequently fail in such settings because they cannot rapidly adapt to the wound geometry or provide sufficiently strong clot stabilization in the face of high-pressure bleeding [13,14]. Consequently, there is an urgent clinical demand for a new generation of hemostatic agents that can be delivered into deep and narrow injury sites and efficiently curtail blood loss.

Injectable hydrogels have attracted substantial attention as emergency hemostatic platforms because they can flow into irregular cavities and form a mechanical barrier in situ. However, their translation is limited by several critical bottlenecks, including the intrinsic trade-off between fast gelation and robust adhesive network integration [15,16,17,18], limited functionality [19], and cumbersome operational requirements. Bioorthogonal chemistries—especially the azide–alkyne click reaction—have opened new avenues for solving these problems [20,21,22,23]. Because of their small size, excellent biocompatibility [24,25], and superb chemoselectivity, azide and alkyne (or strained alkyne) motifs can be incorporated into biomacromolecules without compromising bioactivity, enabling completely specific crosslinking under mild physiological conditions [26,27,28]. Moreover, the mutual orthogonality of these groups permits simultaneous network formation [29] and covalent tethering of supplementary therapeutic modules [30], presenting an elegant strategy for constructing multifunctional hydrogels [31,32,33].

**Scheme 1:**
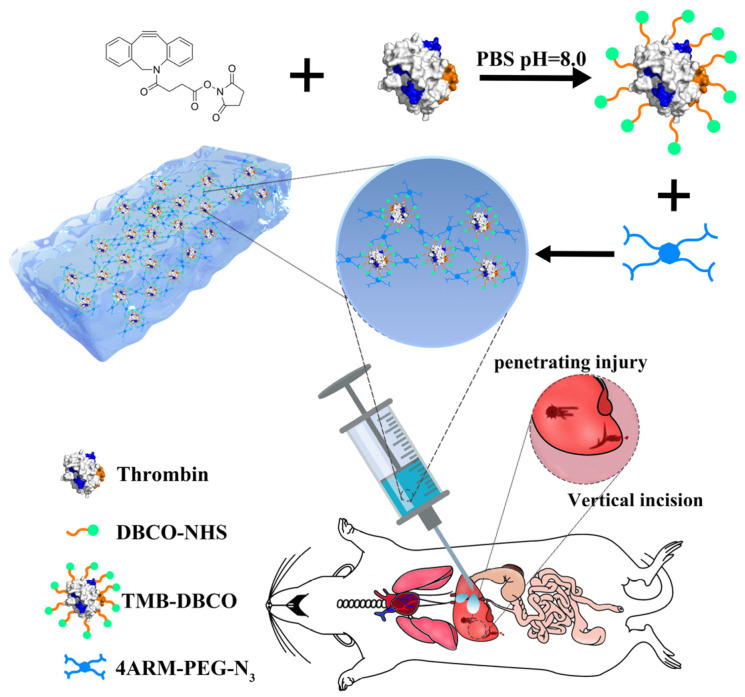
Schematic diagram of the construction and mechanism of the TMB-PEG hydrogel.

Herein, we report an injectable polyethylene glycol hydrogel fabricated through bioorthogonal conjugation between thrombin (TMB) and 4-arm PEG-azide (Scheme 1). Thrombin was first functionalized with dibenzocyclooctyne (DBCO) groups via amide coupling, and subsequent strain promoted azide–alkyne cycloaddition with 4ARM-PEG-N_3_ yielded the TMB-PEG hydrogel. The resulting material exhibited a transparent, injectable character and a hierarchical nanostructure of interwoven fibers. The mild construction method and the biocompatible gel network microenvironment greatly preserve the hemostatic activity of thrombin. Furthermore, cephalosporin antibiotics were conjugated onto the gel through the same orthogonal reaction, conferring broad spectrum antibacterial activity. This modular platform seamlessly integrates rapid hemostasis with infection prevention, offering a facile and potent strategy for managing deep penetrating trauma.

## 2. Results and Discussion

### 2.1. Characterization of the TMB-PEG Hydrogel

**Figure 1:**
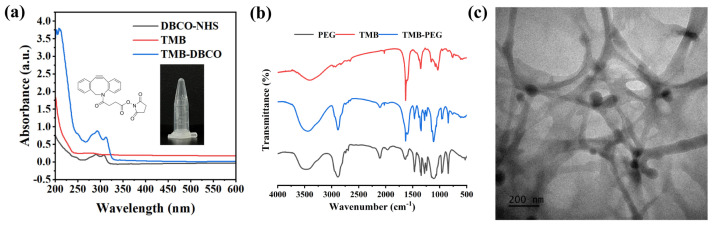
(**a**) UV−vis absorption spectra of TMB, DBCO−NHS, and TMB−DBCO; (**b**) FTIR spectra of PEG, TMB, and TMB−PEG; (**c**) TEM image of TMB−PEG hydrogel.

**Figure 2:**
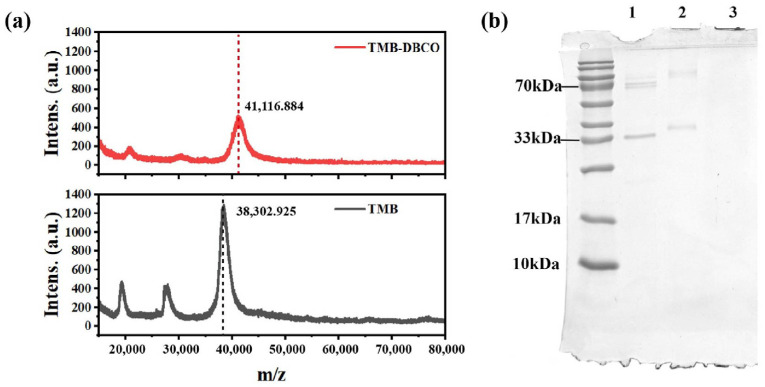
(**a**) MALDI-TOF mass spectra of TMB and TMB-DBCO; (**b**) electrophoresis gel image, with lanes 1–3 corresponding to TMB, TMB-DBCO, and TMB-PEG.

Ultraviolet–visible (UV-vis) absorption spectroscopy was employed to verify the successful conjugation of DBCO moieties with thrombin (TMB). As shown in Figure 1a, the absorption spectrum of native thrombin is primarily attributed to its aromatic amino acid residues, with maximum absorption wavelengths around 280 nm. In contrast, DBCO-NHS exhibits two exclusive characteristic absorption peaks at 290 nm and 310 nm, serving as signature identifiers of the DBCO moiety. The coexistence of thrombin- and DBCO-characteristic peaks in the conjugated product confirmed the successful conjugation of DBCO groups to thrombin. The DBCO modification was also quantified with matrix-assisted laser desorption/ionization time-of-flight mass spectrometry (MALDI-TOF MS). As shown in Figure 2a, the molecular weight of native thrombin (TMB) was measured to be 38,302.925, while the molecular weight of DBCO-modified TMB (TMB-DBCO) was 41,116.884. According to the calculation results, approximately 7 equivalents of DBCO moieties were successfully conjugated onto each thrombin molecule. Meanwhile, in the Fourier transform infrared (FTIR) absorption spectrum (Figure 1b), the absorption peaks in the range of 2850–2960 cm^−1^ were attributed to the stretching vibration of C-H bonds. The amide bond typically exhibits a characteristic peak in the wavenumber range of 1650–1750 cm^−1^, which is associated with the vibration of the oxygen-containing amide group in the amide bond, and is commonly referred to as the amide I band. The presence of all these characteristic peaks confirmed the successful crosslinking between TMB and PEG. The preparation processes were also investigated with the sodium dodecyl sulfate–polyacrylamide gel electrophoresis (SDS-PAGE). The results showed that the molecular weight of TMB-DBCO was slightly higher than that of TMB, while TMB- PEG was trapped in the sample well during electrophoretic migration due to its excessively high molecular weight (Figure 2b). Finally, a clear crosslinked network structure of TMB-PEG could be observed from the transmission electron microscopy (TEM) image (Figure 1c). The hydrogel comprised sheet-like structures and interwoven fibers with a diameter of approximately 100 nanometers.

To further investigate the azide–alkyne cycloaddition (SPAAC) that occurs during hydrogel formation at the molecular level, we characterized TMB-DBCO, 4-arm-PEG-N_3_, and the TMB-PEG hydrogel using ^1^H-NMR. As shown in Figure S1, compared with the individual TMB-DBCO and 4-arm-PEG-N_3_, TMB-PEG exhibits significant changes. In the ^1^H-NMR spectrum of TMB-DBCO, distinct signals are observed at approximately δ 5.1 (a clear multiplet attributed to anomeric protons of glycan residues on the glycoprotein) and δ 1.85 (a weaker multiplet from aliphatic side-chain protons of the protein). However, in the TMB-PEG hydrogel, the characteristic protein peaks at δ 5.1 and 1.85 disappear, which is a direct consequence of the thrombin molecules being covalently crosslinked into the rigid, high-molecular-weight hydrogel network; the severe restriction of molecular tumbling drastically broadens these signals beyond detection. Simultaneously, two new weak multiplets emerge: one at δ 5.75, corresponding to a newly formed olefinic/cyclooctene proton in the triazole-fused cyclooctene ring; and another at δ 7.85, which is the signature of the olefinic proton on the newly created 1,2,3-triazole ring. These signals are only visible from mobile fractions and are definitive spectroscopic fingerprints of the SPAAC ligation between DBCO and azide. The disappearance of the native protein signals together with the appearance of these cycloaddition-specific proton resonances unequivocally confirms that a chemical crosslinking reaction has taken place, successfully forming the TMB-PEG hydrogel network.

Furthermore, to directly demonstrate the contribution of molecular crosslinking to hydrogel formation, two control experiments were performed (Figure S2). Native thrombin (without DBCO modification) was incubated with 4-arm-PEG-N_3_, and DBCO-modified thrombin was incubated with PEG-lacking azide groups. No gelation was observed in either control group. These results confirm that the simultaneous presence of both DBCO and azide (N_3_) is essential for hydrogel formation.

We also evaluated the swelling behavior of the TMB-PEG hydrogel. The hydrogel exhibited significant water-swelling behavior. In the first three hours, its volume expanded by more than 50%. By 6 h, it approached saturation with a swelling ratio of approximately 85%, indicating good liquid absorption capacity of the hydrogel (Figure S3). The biodegradation performance was assessed by incubating the hydrogel with a typical protease trypsin at 37 °C to mimic the biological environment. As shown in Figure S4, the hydrogel degraded significantly in trypsin, and the gel morphology completely disappeared within 6 h. This is attributed to the degradation of thrombin, a key crosslinking point in the hydrogel, by protease, thereby disrupting the gel structure.

In addition, we also characterized the rheology of TMB-PEG hydrogel using a TA Discovery HR 20 hybrid rheometer. As shown in Figure S5, the shear stress increases continuously with increasing shear rate, showing typical features of pseudoplastic fluids. In addition, TMB-PEG hydrogel has a distinct linear viscoelastic region (LVR), in which the storage modulus G′ is always higher than the loss modulus G′′ and remains relatively constant, indicating that the gel network structure is stable and intact under small deformation conditions. After the strain exceeds the critical value, G′ begins to decrease, indicating strain-induced network disruption. At a strain of about 398%, G′ and G′′cross (G′ = G′′), which marks the transition from the gel state to the sol state.

### 2.2. Biocompatibility Evaluation

**Figure 3:**
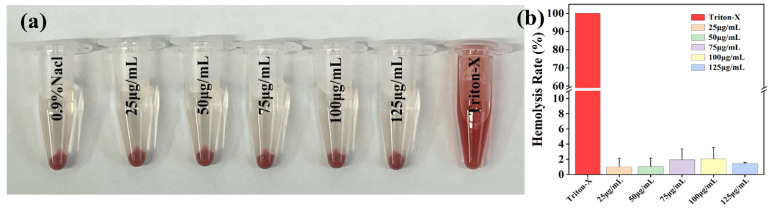
(**a**) Photo of hemolysis experiment; (**b**) TMB-PEG hemolysis rate.

Subsequently, the hemocompatibility of the TMB-PEG hydrogel was evaluated using a hemolysis assay. Hemolysis is defined as the rupture or destruction of red blood cells (RBCs), leading to the release of hemoglobin and other intracellular contents into the surrounding medium. After the incubation, blood samples from each group were centrifuged at 1000× *g* for 3 min, and the occurrence of hemolysis was visually assessed. In the positive control group treated with Triton X-100, the supernatant appeared as a clear, bright red solution with no visible cell pellet at the bottom, unequivocally indicating complete hemolysis (Figure 3). In sharp contrast, the hydrogel-treated test groups exhibited a markedly different behavior: intact RBCs sedimented completely, leaving a clear and colorless supernatant. This observed phenotype confirmed that no obvious hemolysis had occurred in the test groups, and the RBCs maintained their structural integrity. The stark contrast between the positive control and the hydrogel-treated samples validated the reliability of the assay, demonstrating that RBCs in the test groups retained normal membrane architecture without undergoing erythrocyte lysis. To assess the cytotoxicity of TMB-PEG, TMB-PEG hydrogels with or without cephexin were incubated with cells for 24 h, and their biocompatibility was further assessed by live/dead staining. As shown in Figure S6, the TMB-PEG hydrogel showed predominantly green fluorescence (live cells) and least red fluorescence (dead cells) with or without cephexin antibiotics, indicating that the hydrogel formulation did not induce significant cytotoxicity. Furthermore, in our previous experiments, we collected tissue samples from the healed incision and puncture wounds after TMB-PEG treatment, and performed sectioning followed by H&E staining. The results (Figure S7) revealed that in the untreated liver sections from mice with incision and puncture wounds, incompletely healed wound morphology was observed, along with pronounced inflammatory cell infiltration around the wounds. In contrast, the liver wounds in mice treated with the TMB-PEG hydrogel were almost completely healed, and no significant inflammatory infiltration was observed. These findings indicate that TMB-PEG can not only significantly promote wound healing, but also markedly reduce inflammatory infiltration at the wound site.

### 2.3. Extracellular Clotting Performance

**Figure 4:**
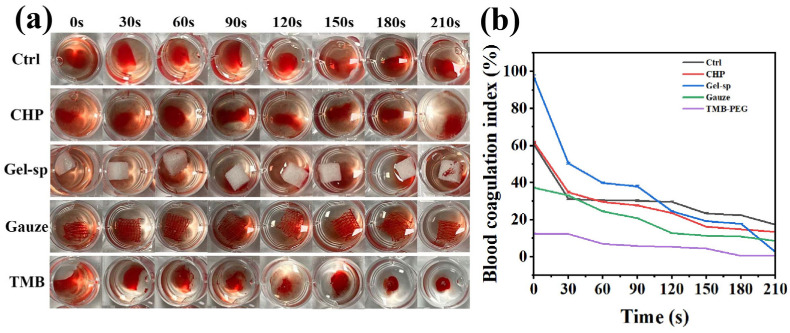
(**a**) Pictures of in vitro coagulation; (**b**) corresponding BCI.

To investigate the in vitro coagulation capacity of the TMB-PEG hydrogel, we evaluated its procoagulant performance using recalcified citrate-anticoagulated whole blood with different commercial hemostatic materials as controls (Figure 4a). Recalcified whole blood in the blank control group coagulated completely at 210 s. The commercial gelatin sponge (Gel-sp) group exhibited significantly inferior hemostatic performance within 120 s due to its limited fluid absorption. The chitosan hemostatic powder (CHP) group showed no significant difference in procoagulant effect compared with the blank control, and the resulting clots were loosely structured and mechanically unstable. The hemostatic gauze group displayed moderate absorption and essentially achieved coagulation by 180 s. In contrast, when the TMB-PEG hydrogel was pre-coated in the wells and recalcified blood was added to the hydrogel surface, rapid clot formation was observed, with the BCI dropping significantly within 30 s. The formed blood clots were compact and remained largely undisturbed during subsequent handling. The blood coagulation index (BCI) of TMB-PEG hydrogel was approximately 10% at the 30 s and approached 0 after the 180 s, which was significantly lower than that of commercial hemostatic materials, indicating that TMB-PEG has more excellent coagulation performance (Figure 4b). By comparing TMB-PEG injectable hydrogel with the existing injectable hydrogels, we prepared an injectable gelatin hemostatic hydrogel using gelatin and conducted a comparative study on its in vitro hemostatic performance. The results (Figure S8) showed that the gelatin hemostatic hydrogel also displayed appreciable hemostatic performance, but it was inferior to the TMB-PEG hemostatic hydrogel developed in this study, further demonstrating the superior hemostatic efficacy of the TMB-PEG hydrogel.

### 2.4. In Vivo Hemostatic Performance

**Figure 5:**
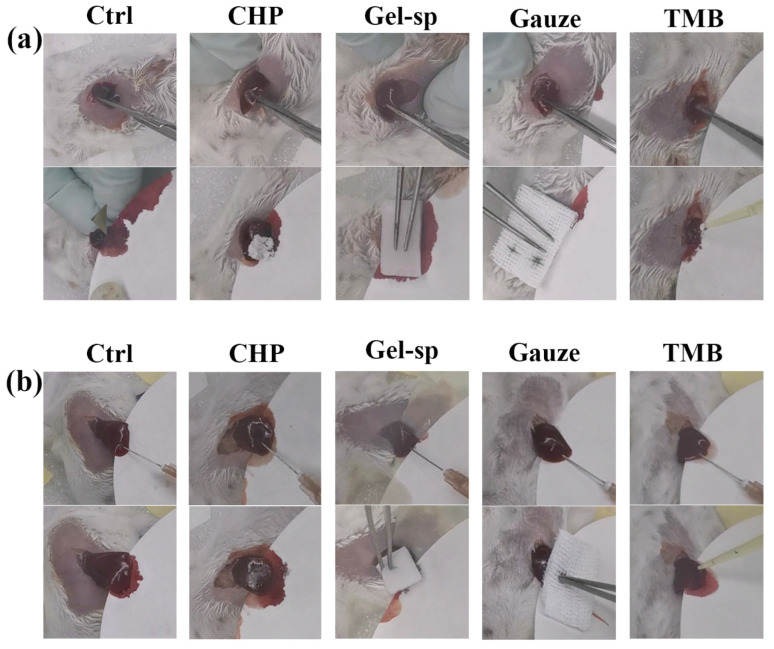
(**a**) Longitudinal shear model of mouse liver edge; (**b**) liver puncture model of mouse.

**Figure 6:**
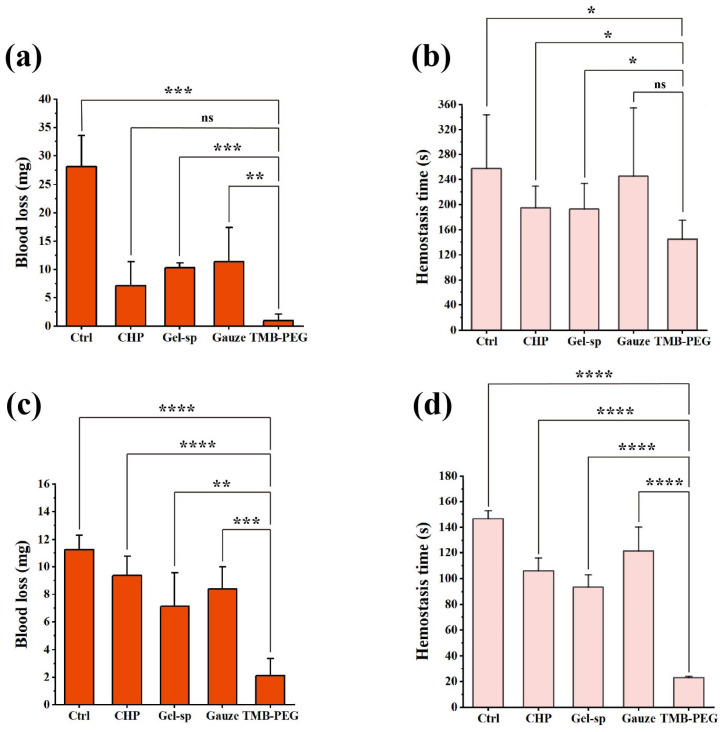
(**a**) Blood loss from laceration injury; (**b**) time for hemostasis of laceration injury; (**c**) blood loss from puncture injury; (**d**) time for hemostasis of puncture injury. Data are presented as mean ± SD (n = 5). Independent sample *t*-tests were used; * = *p* < 0.05, ** = *p* < 0.01, *** = *p* < 0.001, **** = *p* < 0.0001, ns = not statistically significant.

To evaluate the hemostatic efficacy of the TMB-PEG hydrogel for visceral and deep penetrating wounds, we established two mouse liver injury models: the liver longitudinal incision model and the liver puncture model (Figure 5). As shown in Figure 5a and Figure 6a, in the liver longitudinal incision model, the hemostasis time quantification revealed that spontaneous hemostasis in the blank control group required 257 s, compared with 195 s for chitosan powder, 193 s for gelatin sponge, 245 s for sterile gauze, and only 145 s for the TMB-PEG hydrogel. Blood loss measurements showed that the average total blood loss in the control group was 28 ± 7 mg, whereas the TMB-PEG hydrogel reduced blood loss to approximately 1/30 of the control value (1.2 ± 1.3 mg). The blood loss was 7.4 ± 5 mg for chitosan powder, 10.5 ± 1.1 mg for gelatin sponge, and 11.3 ± 7.1 mg for sterile gauze. In the liver puncture injury model (Figure 5b and Figure 6b), the hemostasis time quantification showed that spontaneous hemostasis in the blank control group required 145 s, compared with 110 s for chitosan powder, 93 s for gelatin sponge, 130 s for sterile gauze, and only 25 s for the TMB-PEG hydrogel. Blood loss measurements indicated an average total blood loss of 11 ± 1.1 mg in the control group, while the TMB-PEG hydrogel reduced blood loss to approximately 1/6 of the control value (1.9 ± 1.3 mg). Blood loss values were 9.2 ± 1.5 mg for chitosan powder, 7 ± 2.4 mg for gelatin sponge, and 8.2 ± 1.4 mg for sterile gauze. These results indicate that, in both models, TMB-PEG not only achieves rapid hemostasis but also significantly reduces wound blood loss, with hemostatic performance markedly superior to the three other commercial hemostatic materials. In addition to the pronounced coagulation effect of TMB-PEG, its excellent hemostatic performance in animal wound models also benefits from its injectable, flowable state, which facilitates rapid and full contact with the wound to exert its effect in the shortest possible time.

To further visually demonstrate the application effect of TMB-PEG hydrogel on various irregular wounds, we constructed a gelatinous agar model. As shown in Figure S9, the TMB-PEG hydrogel doped with rhodamine can completely fill the irregular cavities in the agarose gel and even completely penetrate into the narrow gaps. These results clearly demonstrate the broad applicability and advantages of TMB-PEG for irregular wounds.

### 2.5. Inflammatory Factor Expression Levels

**Figure 7:**
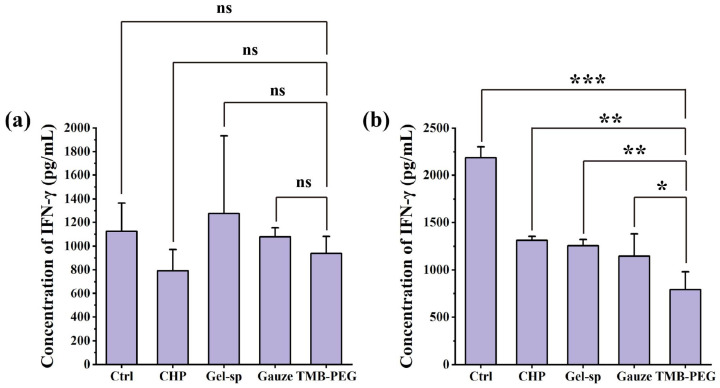
IFN-γ content in serum. (**a**) Serum of mice with longitudinal incision injury; (**b**) serum of mice with puncture injury. Data are presented as mean ± SD (n = 5). Independent sample *t*-tests were used; * = *p* < 0.05, ** = *p* < 0.01, *** = *p* < 0.001, ns = not statistically significant.

**Figure 8:**
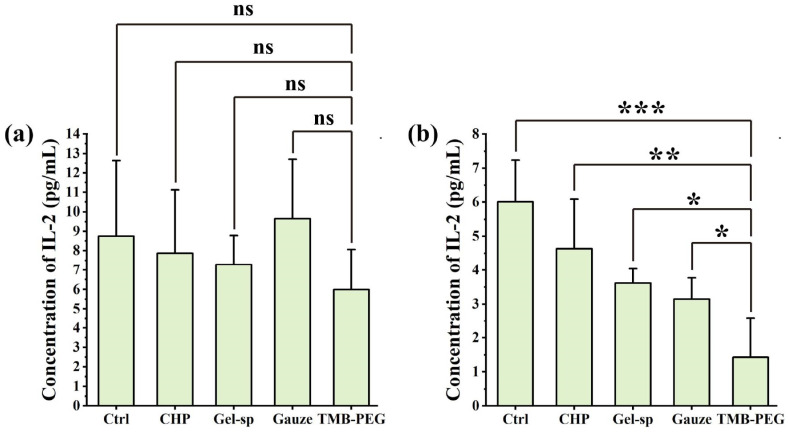
IL-2 content in serum. (**a**) Serum of mice in the incision injury group; (**b**) Serum of mice in the puncture injury group. Data are presented as mean ± SD (n = 5). Independent sample *t*-tests were used; * = *p* < 0.05, ** = *p* < 0.01, *** = *p* < 0.001, ns = not statistically significant.

After hemostatic treatment, we evaluated the effect of treatment on the immune response in mice by measuring two inflammatory cytokines: mouse interferon-γ (IFN-γ) and interleukin-2 (IL-2). The expression levels IFN-γ and IL-2 in serum samples were determined using commercial mouse IFN-γ and IL-2 quantitative detection kits, which were established based on the double-antibody sandwich ELISA method. As shown in Figure 7a and Figure 8a, in serum samples collected from mice in the liver longitudinal incision injury model, IFN-γ and IL-2 concentrations did not show as significant from each other. However, in serum samples from mice in the liver puncture injury model (Figure 7b and Figure 8b), the levels of both IFN-γ and IL-2 in the TMB-PEG group were lower than those in the blank control group and other material-treated groups. These results indicate that TMB-PEG hydrogel can reduce the inflammatory factors induced by liver injury during hemostasis, especially in the liver puncture injury model.

### 2.6. Antibacterial Activity of Antibacterial-Functionalized Hemostatic Hydrogels

**Figure 9:**
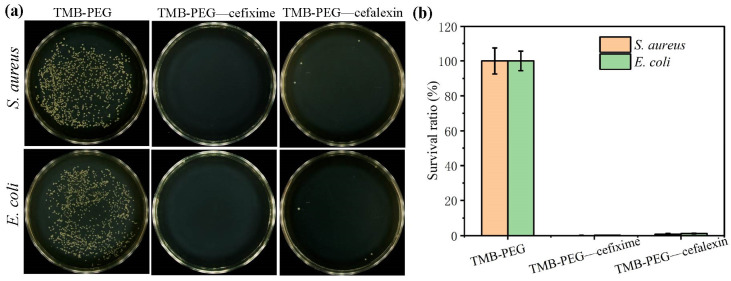
(**a**) Imaging diagrams of the antibacterial activities of different gels; (**b**) statistical chart of bacterial survival rate in antibacterial experiments with different gel treatments.

To further endow the TMB-PEG hemostatic gel with antibacterial activity, we modified the gel with cephalosporin antibiotics using an orthogonal conjugation strategy. The antibacterial activities of the resulting antibacterial hemostatic gels, TMB-PEG–cefixime and TMB-PEG–cefalexin, are shown in Figure 9. As shown in Figure 9a, a large number of bacterial colonies were observed on agar plates co-cultured with TMB-PEG hydrogel, indicating that the hemostatic hydrogel possessed no obvious antibacterial activity. In contrast, nearly no bacterial colonies were detected on the plates of cefixime-functionalized and cefalexin-functionalized hydrogels, which exhibited outstanding antibacterial performance with antibacterial rates higher than 99% (Figure 9b). Such excellent broad-spectrum antibacterial activity enables the injectable hemostatic hydrogel to efficiently prevent wound infection while achieving rapid hemostasis, thereby holding great promise for extensive clinical applications.

After that, we investigated the durable antibacterial activity of the hydrogels. In the experiment, the hydrogels recovered from the initial antibacterial assay were subjected to a second round of antibacterial testing. The results showed that the recovered hydrogels still exhibited significant antibacterial activity. Specifically, the cefixime-modified hydrogel maintained antibacterial rates exceeding 99% against both *E. coli* and *S. aureus*, while the cephalexin-modified hydrogel retained an antibacterial rate of approximately 97%, albeit slightly lower than that of the fresh hydrogel. These findings demonstrate that the hydrogels prepared in this study possess appreciable durable antibacterial activity, as shown in Figure S10. Given that the hemostatic function of the injectable hydrogel is disposable in hemostatic therapy, we did not evaluate its reusable hemostatic activity.

## 3. Conclusions

In this study, an injectable hydrogel with high-efficiency hemostatic performance was fabricated via a bioorthogonal coupling reaction between the thrombin and 4ARM-PEG. The resulting hydrogel exhibited markedly better hemostatic capacity than multiple commercially available hemostatic materials. Benefiting from its injectable property the hydrogel realized rapid hemostasis for deep penetrating wounds. Furthermore, cephalosporin antibiotics were covalently conjugated through bioorthogonal reactions, endowing the hemostatic hydrogel with excellent broad-spectrum antibacterial activity. In conclusion, this work established a facile strategy for the fabrication of multifunctional antibacterial and hemostatic hydrogels, which possesse great potential for clinical translation and practical application in emergency trauma treatment.

## 4. Materials and Methods

### 4.1. Preparation of TMB-PEG Hydrogel

TMB was first modified with DBCO through the reaction with DBCO-NHS. A 40 mM stock solution of dibenzocyclooctyne N-hydroxysuccinimide ester (DBCO-NHS) was prepared in dimethyl sulfoxide (DMSO). The TMB solution was mixed thoroughly with the DBCO-NHS stock solution at a TMB:DBCO-NHS molar ratio of 1:50. Phosphate-buffered saline (PBS, pH 8.0) was then added to bring the final concentration of DBCO-NHS to 10 mM, and the mixture was stirred overnight at room temperature. Unreacted DBCO-NHS was removed by ultrafiltration using a 10 kDa MWCO centrifugal filter, and the TMB-DBCO conjugate was concentrated and collected. After this, a 100 mg/mL solution of 4-arm poly(ethylene glycol) azide (4ARM-PEG-N_3_) was prepared in deionized water. This solution was mixed with the TMB-DBCO conjugate to form the TMB-PEG hydrogel.

### 4.2. Characterization of the TMB-PEG Hydrogel

UV-vis absorption spectra of DBCO-NHS, TMB, and TMB-DBCO were recorded over 200–800 nm on a UV-vis spectrophotometer (Thermo Fisher Scientific, Radnor, PA, USA). Fourier transform infrared (FTIR) spectroscopy was performed from 4000 to 500 cm^−1^ to characterize changes in functional groups of TMB-DBCO before and after crosslinking. Gel electrophoresis was performed to qualitatively analyze the molecular weights of TMB, DBCO-TMB, and TMB-PEG. The molecular weights of TMB and DBCO-TMB were determined by matrix-assisted laser desorption/ionization time-of-flight (MALDI-TOF) mass spectrometry to confirm the covalent conjugation between TMB and DBCO-NHS. The rheology of TMB-PEG hydrogel was measured by a TA Discovery HR 20 hybrid rheometer (TA Instruments, New Castle, DE, USA).

### 4.3. Cytotoxicity Assay

Hydrogels with or without antibiotic supplementation were uniformly coated onto the bottom of 96-well plates. Wells without hydrogel coating served as the control group. 4T1 cells in the logarithmic growth phase were seeded onto the hydrogel-coated wells at a density of 5000 cells per well. After incubation at 37 °C for 24 h, the culture medium was gently removed, and 100 μL of fresh complete medium was added to each well. Subse-quently, the staining working solution was prepared according to the instructions using the Calcein/PI Cytotoxicity Assay Kit (C2015S, Beyotime, Shanghai, China). Then, 100 μL of the staining solution was added to each well, and the cells were incubated at 37 °C for 30 min in the dark. Finally, fluorescent images were acquired using an NCF950 confocal micro-scope (Yongxin Optics Co., Ltd., Ningbo, China), where green fluorescence (Calcein-AM) indicated live cells and red fluorescence (PI) indicated dead cells.

### 4.4. Hemolysis Assay

Commercial rabbit whole blood was purchased for the hemolysis test. An approximate 10-fold volume of 0.9% (*w*/*v*) NaCl solution was added to the whole blood, mixed thoroughly, and centrifuged at 150× *g* for 15 min. After discarding the supernatant, the precipitated erythrocytes were washed three times with 0.9% NaCl solution following the same procedure until the supernatant was colorless. The washed erythrocytes were resuspended in 0.9% NaCl solution to prepare a 4% (*v*/*v*) erythrocyte suspension.

Serial working solutions of the test samples were prepared at concentrations of 125, 100, 75, 50, and 25 μg/mL. The positive control was set as 2% (*v*/*v*) Triton X-100. Each test sample or positive control was mixed with an equal volume of the 4% erythrocyte suspension. The negative control was prepared by mixing equal volumes of the 4% erythrocyte suspension and 0.9% NaCl solution.

All mixtures were incubated at 37 °C with shaking at 60 rpm for 1 h, followed by centrifugation at 1000× *g* for 3 min. An aliquot of 100 μL the supernatant was carefully transferred to a 96-well plate, and the absorbance was measured at 540 nm.

The hemolysis rate was calculated as follows:

(1) $$\;Hemolysis\;rate\;(\%)=\frac{OD_{sample\;group}-OD_{negativecontrol}}{OD_{positivecontrol}-OD_{negativecontrol}}\times 100\;\%$$

### 4.5. In Vitro Blood Coagulation Assay

A total of 0.2 M CaCl_2_ working solution was prepared for the recalcification of sodium citrate-anticoagulated commercial rabbit whole blood, with the final concentration of CaCl_2_ in the blood adjusted to 20 mM. Commercial chitosan hemostatic powder, gelatin sponge, sterile gauze, and TMB-PEG hydrogel were separately placed in a 24-well plate and pre-warmed at 37 °C, with wells without any hemostatic material set as the blank control group. A total of 20 µL of the recalcified whole blood (with a final CaCl_2_ concentration of 20 mM obtained by adding the 0.2 M CaCl_2_ working solution) was then slowly dropped onto the surface of the pre-warmed materials in each well or the blank control wells.

The plate containing blood samples was placed in a constant temperature incubator and incubated at 37 °C for 0, 30, 60, 90, 120, 150, 180 and 210 s. At each preset time point, 2 mL of deionized water was gently added to release uncoagulated blood without disrupting the formed blood clots. The absorbance of the supernatant was measured at 545 nm using a microplate reader. Each group was set with three parallel replicate wells, and the experiment was independently repeated three times.

The blood coagulation index (BCI) of the materials was quantified using the following formula:

(2) $$\;BCI=\;\frac{I_{t}-I_{c}}{I_{r}-I_{c}}\times 100\%$$

where *I_t_* is the absorbance of the test sample supernatant; *I_r_* is the absorbance obtained from 20 µL of recalcified whole blood that has been completely hemolyzed in 2 mL of deionized water; and *I_c_* is the absorbance of deionized water alone.

### 4.6. In Vivo Liver Injury Hemostasis Model

The in vivo hemostatic properties of the TMB-PEG hydrogel were evaluated by using a mouse liver hemorrhage model. The animal experiments were approved by the Ethics Committee of Animal Experiments in Zhejiang Sci-Tech University, and all procedures followed the guidelines for animal experiments in Zhejiang Sci-Tech University. Healthy 4-week-old female Kunming (KM) mice were randomly divided into groups (n = 5 per group), weighed, and their body weights recorded. Then, 20% (*w*/*v*) urethane working solution was freshly prepared in autoclaved normal saline and injected intraperitoneally (1000 mg/kg body weight). This anesthetic regimen maintains anesthesia for 2–4 h with low toxicity and is well-suited for small laboratory animals. A small subxiphoid incision was made, and the left lobe of the liver was gently externalized. Two injury models were established: the liver longitudinal incision model—a 5 mm longitudinal incision was made inward from the edge of the left lobe using surgical scissors; and the liver puncture injury model—a deep wound (1 mm diameter, 3 mm depth) was created at the edge of the left lobe using a syringe needle.

After allowing the wound to bleed freely for 10 s, the mice in the blank control group received no treatment, while the experimental groups were treated with chitosan hemostatic powder, gelatin sponge, sterile gauze, and TMB-PEG hydrogel. Pre-weighed, clean, dry filter paper was used to continuously blot the oozing blood. After the hemostasis is completed, the mass of the filter paper is weighed again, and the blood loss in each group is calculated based on the difference in mass between the two weighings. After the surgery, the abdomen of the mice was sutured, and they were kept separately for observation.

### 4.7. Inflammatory Factor Detection

The Enzyme-Linked Immunosorbent Assay (ELISA) is a highly sensitive immunological technique widely used for the quantitative detection of antigens or antibodies in biological samples. The detailed procedure is described as follows:

Blood was collected from mice via retro-orbital puncture into pyrogen- and endotoxin-free tubes. The blood samples were allowed to clot at room temperature and then kept at 4 °C for 4 h to allow clot retraction. Following centrifugation at 4000 rpm for 20 min at 4 °C, the supernatant serum was carefully collected for subsequent analysis.

Liver tissues were rinsed with pre-chilled 0.01 M phosphate-buffered saline (PBS, pH 7.4) to remove residual blood, weighed, and minced. The minced tissue was mixed with pre-chilled PBS at a ratio of 1:9 (*w*/*v*) and thoroughly homogenized on ice using a glass homogenizer. The resulting homogenate was further disrupted by ultrasonication to ensure complete cell lysis. Finally, the homogenate was centrifuged at 5000× *g* for 10 min at 4 °C, and the supernatant was collected as the liver tissue homogenate sample.

For the detection of mouse interleukin-2 (IL-2), 50 μL each of the mouse IL-2 standard, liver tissue homogenate supernatant, and serum sample was respectively added to the wells of a microplate pre-coated with anti-mouse IL-2 capture antibody, followed by the addition of horseradish peroxidase (HRP)-conjugated anti-mouse IL-2 detection antibody. The plate was incubated at 37 °C for 60 min in the dark. After incubation, the liquid in the wells was discarded, and the plate was thoroughly blotted against absorbent paper. Each well was then filled with 0.05% Tween 20 washing solution (diluted 20-fold), allowed to stand for 20 s, and then the washing solution was decanted. The plate was blotted against absorbent paper. This washing step was repeated five times to completely remove unbound components, leaving a solid-phase “capture antibody–antigen–detection antibody” sandwich complex on the well surface.

Subsequently, a substrate solution containing 0.01% hydrogen peroxide and 0.1% 3,3′,5,5′-tetramethylbenzidine (TMB) was added to each well. The HRP-catalyzed reaction produced a blue product, which was converted to a stable yellow color upon the addition of stop solution (2 M H_2_SO_4_). The absorbance (OD value) of each well was measured at 450 nm using a microplate reader, with the OD_450_ value being positively correlated with the IL-2 concentration in the sample.

The concentration of IL-2 in each sample was interpolated from the standard curve to reflect the inflammatory status of the mice. The detection procedure for mouse interferon-γ (IFN-γ) was identical to that for IL-2.

### 4.8. Antibacterial Functionalization and Activity of the Hydrogels

Cephalosporin antibiotics (cefixime and cefalexin) were each coupled with DBCO-NHS at a molar ratio of 1:1 to yield DBCO-functionalized cefixime (DBCO–cefixime) and DBCO-functionalized cefalexin (DBCO–cefalexin). DBCO–cefixime and DBCO–cefalexin (final concentration, 1 mM) were then separately mixed with the thrombin–DBCO conjugate (TMB-DBCO) and 4ARM-PEG-N_3_ to fabricate injectable hemostatic/antibacterial hydrogels loaded with cefixime or cefalexin via in situ click crosslinking.

The in vitro antibacterial activity was evaluated using the agar plate colony counting method. Standard strains of Escherichia coli (*E. coli*, ATCC 25922) and Staphylococcus aureus (*S. aureus*, ATCC 25923) were employed as representative Gram-negative and Gram-positive bacteria, respectively. The cryopreserved strains were retrieved from a −80 °C freezer and then streaked onto Luria–Bertani (LB) agar plates for activation. A single colony was inoculated into 5 mL of LB broth and cultured at 37 °C with shaking at 220 rpm until reaching logarithmic growth phase (OD_600_ ≈ 0.6). The bacterial suspension was subsequently diluted with sterile phosphate-buffered saline (PBS) to approximately 1 × 10^7^ CFU/mL.

The prepared cefixime-loaded and cefalexin-loaded antibacterial hydrogels were placed in a 96-well plate. An aliquot of 10 µL of the bacterial suspension was dropped onto the surface of each gel sample to ensure the droplet completely covered the hydrogel surface. The original TMB-PEG hydrogel without cephalosporin antibiotics served as the control. All samples were incubated at 37 °C with ≥90% relative humidity for 3 h. Following incubation, the gel together with the bacterial suspension was transferred to a sterile centrifuge tube, and 100 µL of sterile PBS was added. After thorough mixing, 10-fold serial dilutions were prepared, and 100 µL of the appropriately diluted bacterial suspension was spread onto LB agar plates. Colonies were counted after inverted incubation at 37 °C for 16–18 h.

## Data Availability

The data presented in this study are available on request from the corresponding author.

## Supplementary Materials

The following supporting information can be downloaded at: https://www.mdpi.com/article/10.3390/gels12060556/s1, Figure S1: The 1H-NMR spectra of TMB-DBCO, 4arm-PEG-N3 and TMB-PEG hydrogel. Figure S2: The mixture of TMB and PEG with different modications. Figure S3: The swelling ratio of TMB-PEG hydrogel. Figure S4: Photographs of the degradation of TBM-PEG hydrogel in the presence of trypsin. Figure S5: (a) Curve of shear stress as a function of shear rate for TMB-PEG hydrogel, (b) Oscillatory strain sweep of the TMB-PEG at a constant frequency of 10 rad/s. Storage modulus G′ and loss modulus G″ as a function of oscillatory strain (%). Figure S6: Live/dead staining of cells after incubation with TMB-PEG containing cefamandole antibiotics and TMB-PEG without antibiotics for 24 h. Figure S7: Representative H&E staining images of liver tissue sections from two injury models (longitudinal incision injury and puncture injury) after different treatments. Figure S8: (a) Representative photographs of in vitro blood coagulation on different materials, control group (Ctrl), TMB-PEG hydrogel (TMB), Injectable gelatin hydrogel (IGH), PEG hydrogel (PEG), at various time points (0, 30, 60, 90, 120, 150, 180, 210 s); (b) Blood Coagulation Index (BCI) of different groups measured at time points from 0 to 210 s. Figure S9: The photographs of the agarose gel irregular wound model before (a) and after (b) the TMB-PEG (Rhodamine B doped) injection, and the top view (c). Figure S10: (a) Representative images of agar plates showing colonies of *S. aureus* and *E. coli* after incubation with the recycled and reused hydrogels different hydrogels (TMB-PEG, TMB-PEG-cefixime, and TMB-PEG-cefalexin); (b) Statistical analysis of bacterial survival rates corresponding to (a).

## Abbreviations

The following abbreviations are used in this manuscript:

4ARM-PEG-N_3_	4-arm poly(ethylene glycol) azide
BCI	Blood coagulation index
CHP	Chitosan hemostatic powder
DBCO	Dibenzocyclooctyne
DBCO-NHS	Dibenzocyclooctyne N-hydroxysuccinimide ester
DMSO	Dimethyl sulfoxide
*E. coli*	*Escherichia coli*
ELISA	Enzyme-linked immunosorbent assay
FTIR	Fourier transform infrared
Gel-sp	Gelatin sponge
HRP	Horseradish peroxidase
IFN-γ	Interferon-γ
IL-2	Interleukin-2
KM	Kunming
LB	Luria–Bertani
MALDI-TOF MS	Matrix-assisted laser desorption/ionization time-of-flight mass spectrometry
MWCO	Molecular weight cut-off
OD	Optical density
PBS	Phosphate-buffered saline
PEG	Polyethylene glycol
RBC	Red blood cell
*S. aureus*	*Staphylococcus aureus*
SDS-PAGE	Sodium dodecyl sulfate–polyacrylamide gel electrophoresis
TEM	Transmission electron microscopy
TMB	Thrombin
UV-vis	Ultraviolet–visible
