# Supplementary material for: Bioorthogonally Cross-Linked Injectable PEG Hydrogel with Robust Hemostatic and Antibacterial Properties
Source: Gels. 2026 Jun 20;12(6):556. doi: 10.3390/gels12060556 (PMC13298720; doi:10.3390/gels12060556)
Supplement: Supplementary file 1 [file gels-12-00556-s001.zip › gels-4337927-supplementary.pdf]

Supporting Information

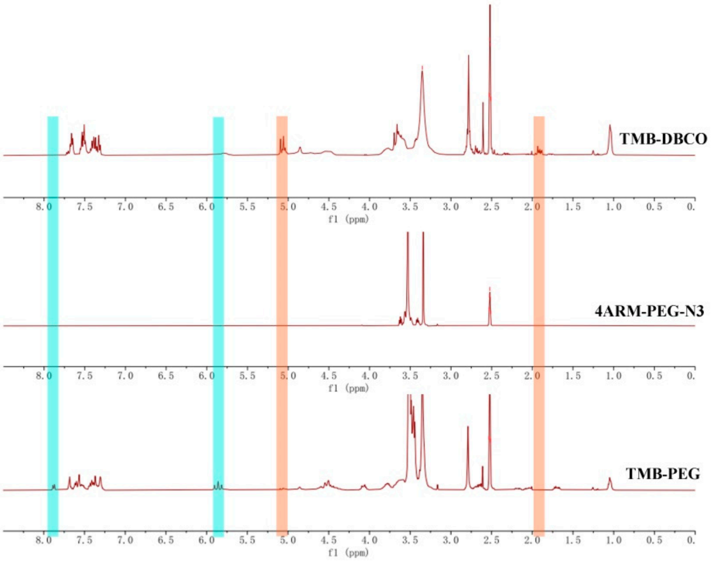

Figure S1 The ^{1}H-NMR spectra of TMB-DBCO, 4arm-PEG-N_3 and TMB-PEG hydrogel.

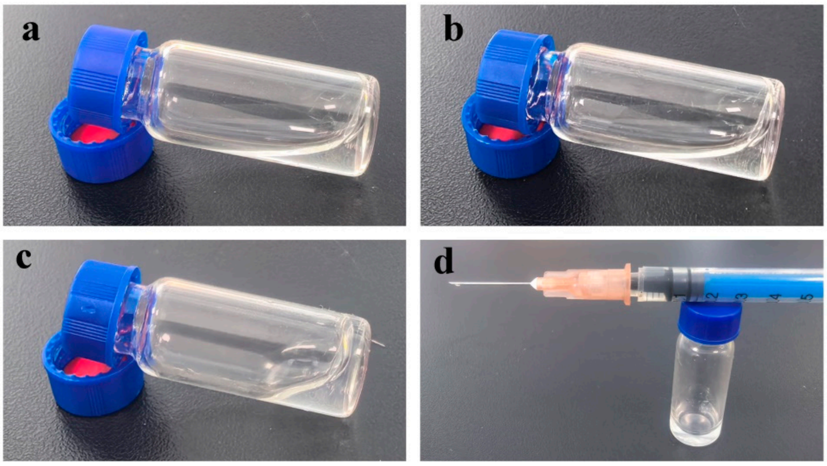

Figure S2 The mixture of TMB and PEG with different modications. a) Native thrombin (without DBCO modification) incubated with 4-arm-PEG-N_3, b) DBCO-modified thrombin (TMB-DBCO) incubated with PEG lacking azide groups (4-arm-PEG), c) TMB-DBCO incubated with 4-arm-PEG-N_3. d) The state of the hydrogel in c being sucked into the syringe.

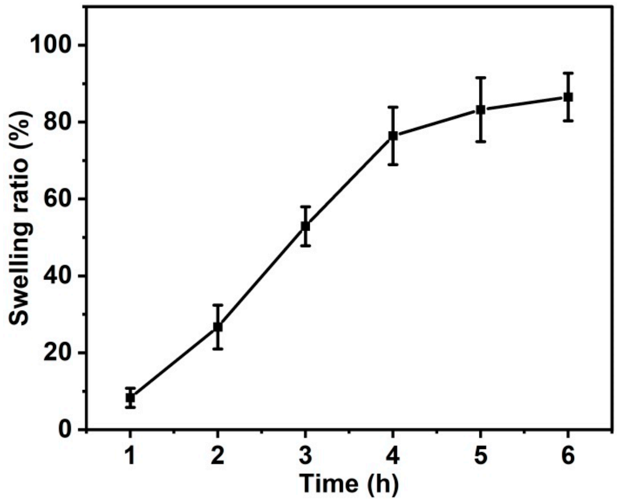

Figure S3 The swelling ratio of TMB-PEG hydrogel.

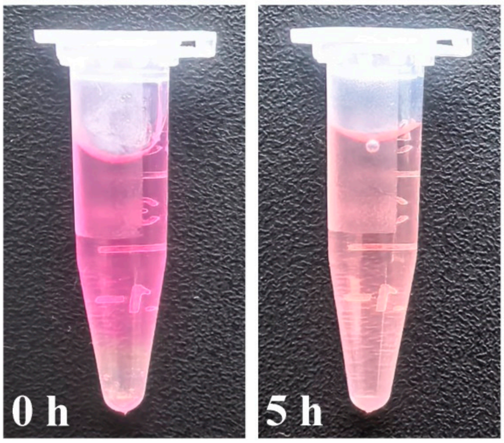

Figure S4 Photographs of the degradation of TBM-PEG hydrogel in the presence of trypsin.

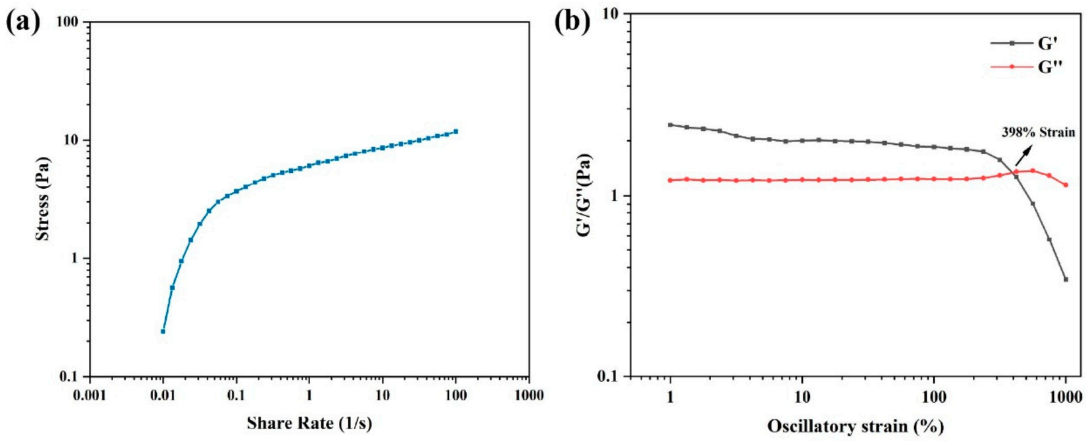

Figure S5 a) Curve of shear stress as a function of shear rate for TMB-PEG hydrogel, **b)** Oscillatory strain sweep of the TMB-PEG at a constant frequency of 10 rad/s. Storage modulus G' and loss modulus G "as a function of oscillatory strain (%).

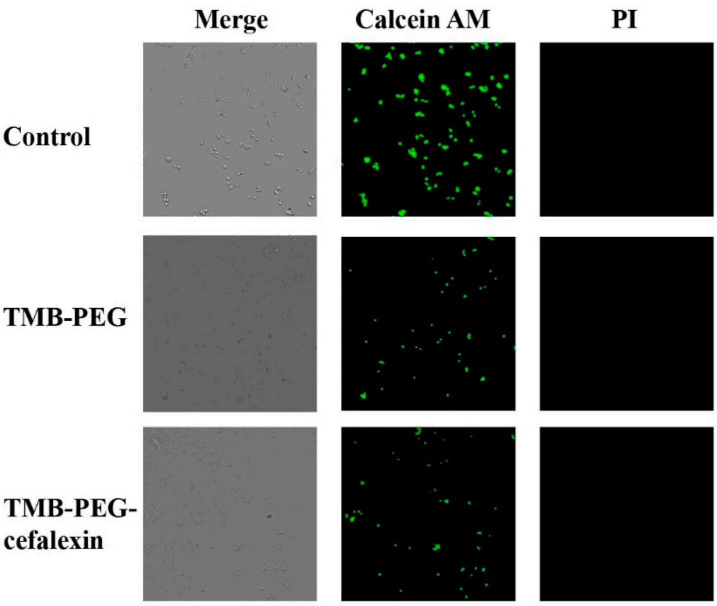

Figure S6 Live/dead staining of cells after incubation with TMB-PEG containing cefamandole antibiotics and TMB-PEG without antibiotics for 24 hours.

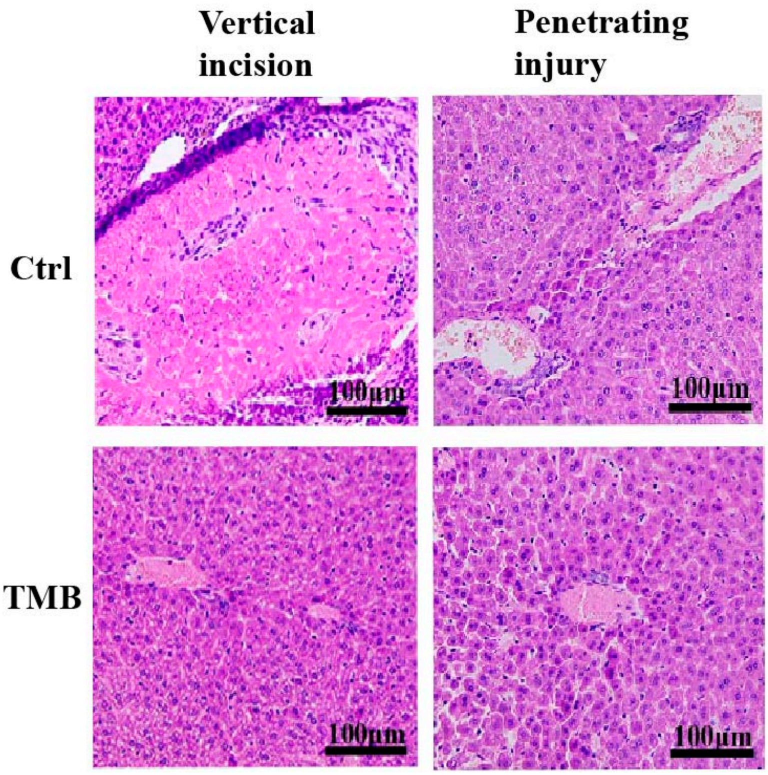

Figure S7 Representative H&E staining images of liver tissue sections from two injury models (longitudinal incision injury and puncture injury) after different treatments. Ctrl: untreated control group; TMB: TMB-PEG hydrogel-treated group. Scale bars = 100 μm.

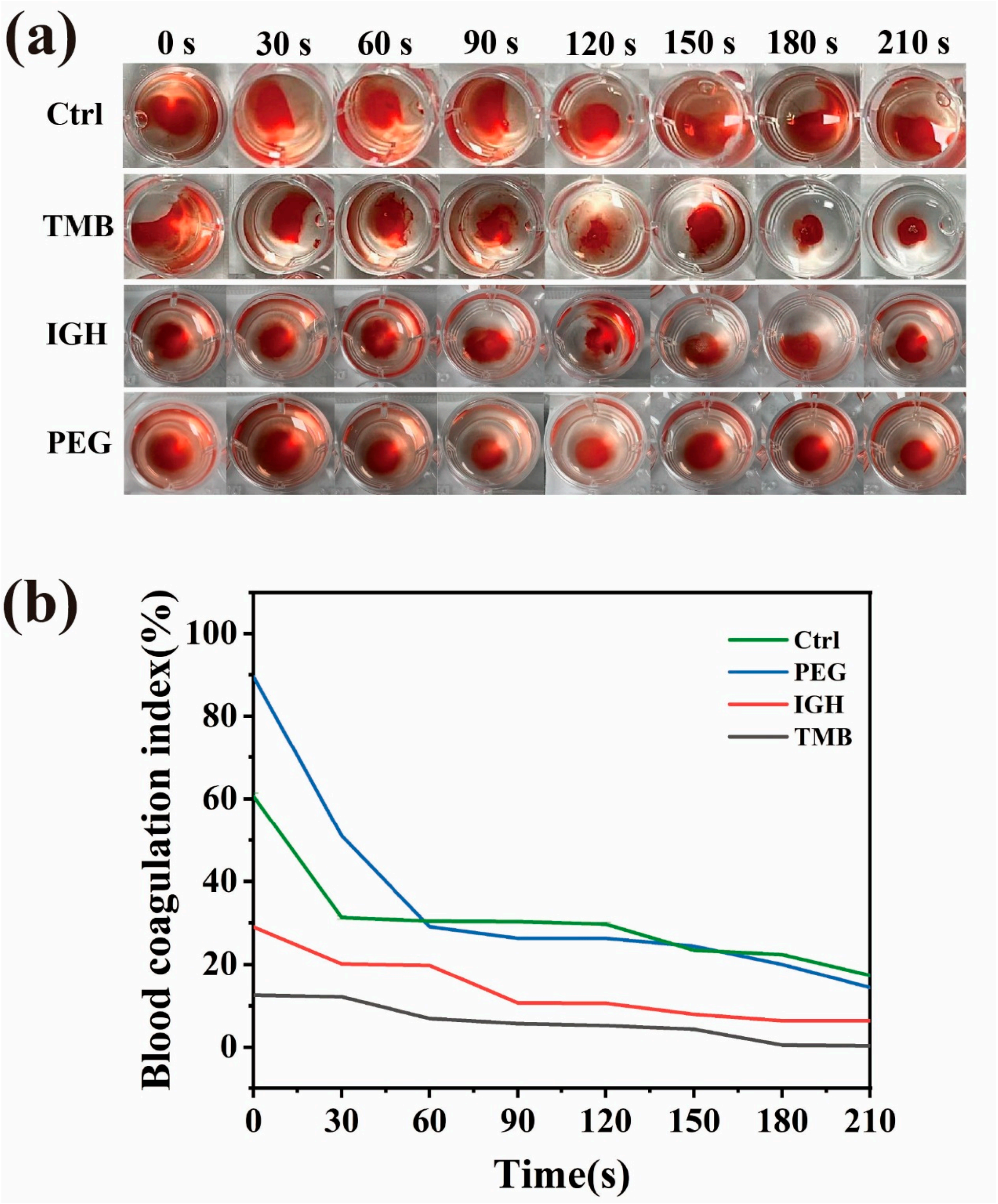

Figure S8 a) Representative photographs of *in vitro* blood coagulation on different materials, control group (Ctrl), TMB-PEG hydrogel (TMB), Injectable gelatin hydrogel (IGH), PEG hydrogel (PEG), at various time points (0, 30, 60, 90, 120, 150, 180, 210 s); **b)** Blood Coagulation Index (BCI) of different groups measured at time points from 0 to 210 seconds.

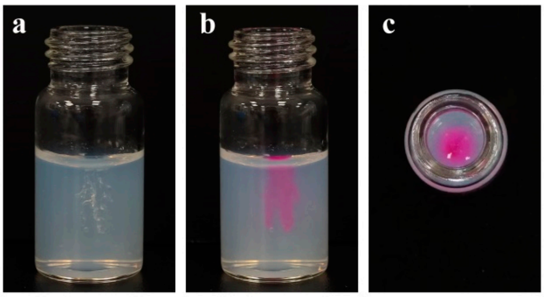

Figure S9 The photographs of the agarose gel irregular wound model before **(a)** and after **(b)** the TMB-PEG (Rhodamine B doped) injection, and the top view **(c)**.

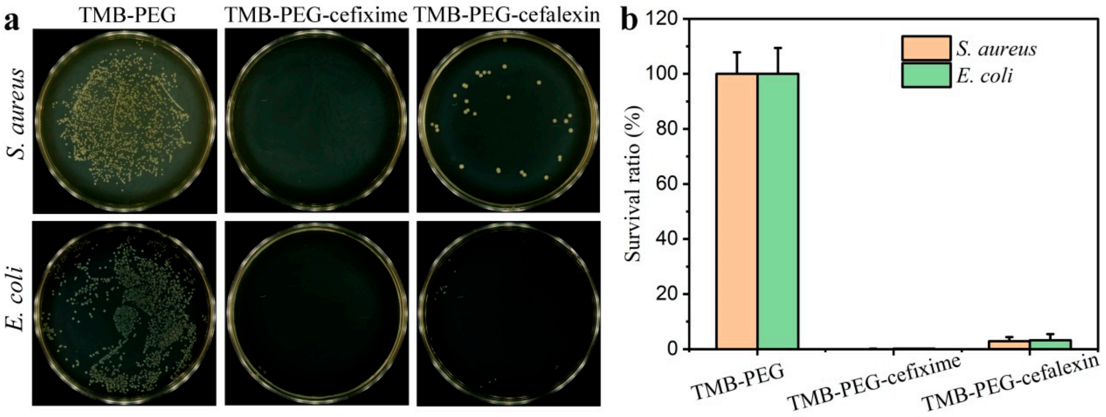

Figure S10 a) Representative images of agar plates showing colonies of *S. aureus* and *E. coli* after incubation with the recycled and reused hydrogels different hydrogels (TMB-PEG, TMB-PEG-cefixime, and TMB-PEG-cefalexin); **b)** Statistical analysis of bacterial survival rates corresponding to (a).
